# Balance and imbalance in dark and bright (OFF and ON) visual channels

**DOI:** 10.1038/s41598-025-28463-y

**Published:** 2026-01-14

**Authors:** Ernest Greene, Jack Morrison

**Affiliations:** Psychophysics Research Laboratory, Department of Psychology, University of Southern California, Los Angeles, CA USA

**Keywords:** Contrast polarity OFF & ON channels Talbot-Plateau anomalous contrast, Neuroscience, Physics

## Abstract

The visual system uses separate neuron channels to distinguish figures that are darker or brighter than their background. We displayed letters as dark and/or bright pulses, with pulse energy scaled as a function of background energy, and assessed the probability of letter recognition as a function of pulse energy and polarity. Similarity of effect for dark and bright pulses was tested with single pulses and with flicker displays. The role of frequency and polarity within flicker cycles was evaluated with reference to provisions of the Talbot-Plateau law. Some energy ranges of dark and bright energy produced comparable changes in the probability of letter recognition, while others did not. The results also provide insight into what stimulus conditions will produce a previously reported “anomalous contrast” effect.

**Supplementary Information:**

The online version contains supplementary material available at 10.1038/s41598-025-28463-y.

## Introduction

*“The most startling of the photo-electric changes in the eye is undoubtedly that which occurs on the cessation of light*,* and which for brevity I term the light off or more tersely the OFF effect. That light should produce a photo-electric change of a given type is to be expected since the eye is framed to react to light rays*,* but that a change of the same type should also be produced by darkness*, i.e.,* the absence of light*,* is remarkable from every point of view.” Francis Gotch 1903 p. 400*^1^.

Though 122 years has passed since Gotch observed a visual response to the cessation of light, a number of issues remain unresolved. We need greater insight into what conditions produce asymmetry of perceptual and neuronal response. Humans respond faster and are more accurate a perceiving dark versus bright transitions^2–7^. Norcia and associates recorded evoked potentials in humans and found faster and higher amplitude evoked potentials for activation by dark relative to bright stimuli^8^. Luo-Li and associates found that orientation discrimination of dark stimuli was faster and required a lower threshold^9^.

The physiological basis for these differentials begins early in the visual system. Numerous studies have reported that retinal neurons are more sensitive and respond faster to dark transitions than to bright transitions^10–15^. However, there are numerous reasons for finding differentials in the perceptual and physiological response to dark and bright stimulation^16^. Further work to see whether there are differences in their ability to elicit discrimination of figures (letters) from background luminance seems warranted.

Further, using dark and/or bright departures from background (pulses) can provide insights about dual-channel (ON and OFF) mechanisms of the visual system that register and signal image contrasts. The Talbot-Plateau law specifies what combination of flicker parameters, i.e., frequency x duration x intensity, will be perceived to be the same brightness as a steady (nonflickering) background^17–19^. If the letters are displayed with Talbot-Plateau combinations that are weighted more heavily with dark-pulse energy, they more likely will be seen as dark letters. Conversely, the pulse combinations more heavily weighted with bright energy are most often seen as bright. Models of these judgments are fairly linear as a function of degree of dark and bright weighting^20^.

Most intriguing, combinations that provide an extreme mismatch of bright and dark pulse durations can violate the Talbot-Plateau predictions by allowing letter recognition that is well above what would be predicted. We have described this as “anomalous contrast”^21^. The anomalous contrast effect can be seen with the weighted combinations described above, but is most dramatic where the dark and bright pulses are predicted to produce luminance that matches the background. Letters being displayed with luminance-balanced combinations should not be visible against the background, yet recognition can be in the 95–100% range.

One goal of the present work was to better assess the relative contribution of pulse frequency, duration, and intensity in producing anomalous contrast. We examined the degree to which dark and bright pulses were comparable when presented as single pulses, or with unipolar flicker, or with bipolar flicker. (Unipolar flicker provides sequencies of dark or bright pulses for a portion of each period, with return to background for the remainder of the period. Bipolar flicker provides sequences that have both dark and bright pulses within each period.) Early pilot work indicated that each display method provided the most consistent data if the quantity of light was scaled as “energy.” This fits well with the Talbot-Plateau law, since (duration x intensity) determines the energy level. We restricted both dark and bright displays to the range of energy that could be produced by dark pulses.

## Methods

### Display equipment

A 64 × 64 array of red LEDs provided the stimulus displays. This display system was custom designed and fabricated by Digital Insight. Peak emission of the LEDs was 633 nm, and rise/fall time of emission is 3 ns. A given LED provides 90% of its light within a span of 5.3 mm, which is designated as the dot diameter. Center-to-center spacing was 4.83 mm and the total span of the array was 309 mm. Viewing distance was 1.5 m, so the visual angles for dot diameter, spacing, and span were 12, 11, and 707 min of visual angle, respectively. The display system has timing precision of at least 1µs. Experimental control of stimulus display and recording of respondent judgments were provided by a compact Windows PC, custom programmed with Tcl/Tk applications.

### Letter design

**Fig. 1:**
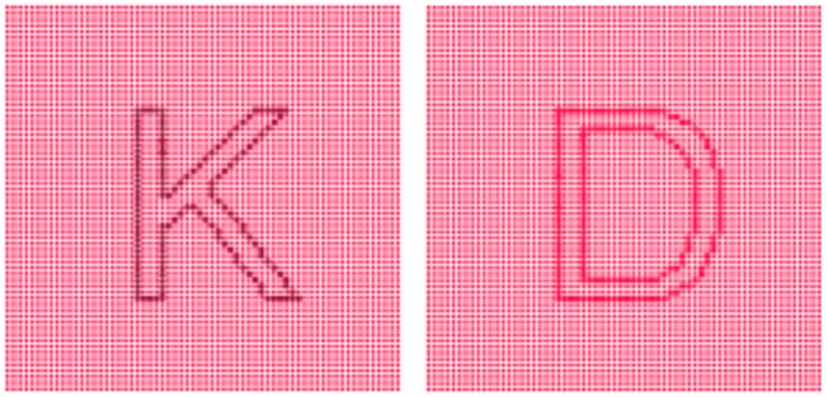
Dots that provided the outline of letters were displayed against a steady level of background luminance. They appeared dark if the pulses provided less energy than background and bright if the pulses energy was greater than background.

Dot patterns provided outline letters, designed like Arial True-Type 60-point fonts, wherein a single continuous string of dots marked the boundaries of the letters. Examples are provided in Fig. 1. Letters were 32 dots tall. The letter outlines could appear dark or bright against a uniform background, or be invisible, depending on treatment conditions.

### Stimulus displays and light energy specifications

Background intensity was 8 Cd/m^2^ for each of the tasks. For convenience, we will use the common term “nits” as a substitute for Cd/m^2^. Letters were displayed as increments or decrements of emission relative to background. Increments and decrements of emission are described as being bright and dark “pulses,” and may also be characterized as having bright and dark “polarity.” (We are avoiding the term “flash,” as many would not view a decrease in light emission as a flash.)

**Fig. 2:**
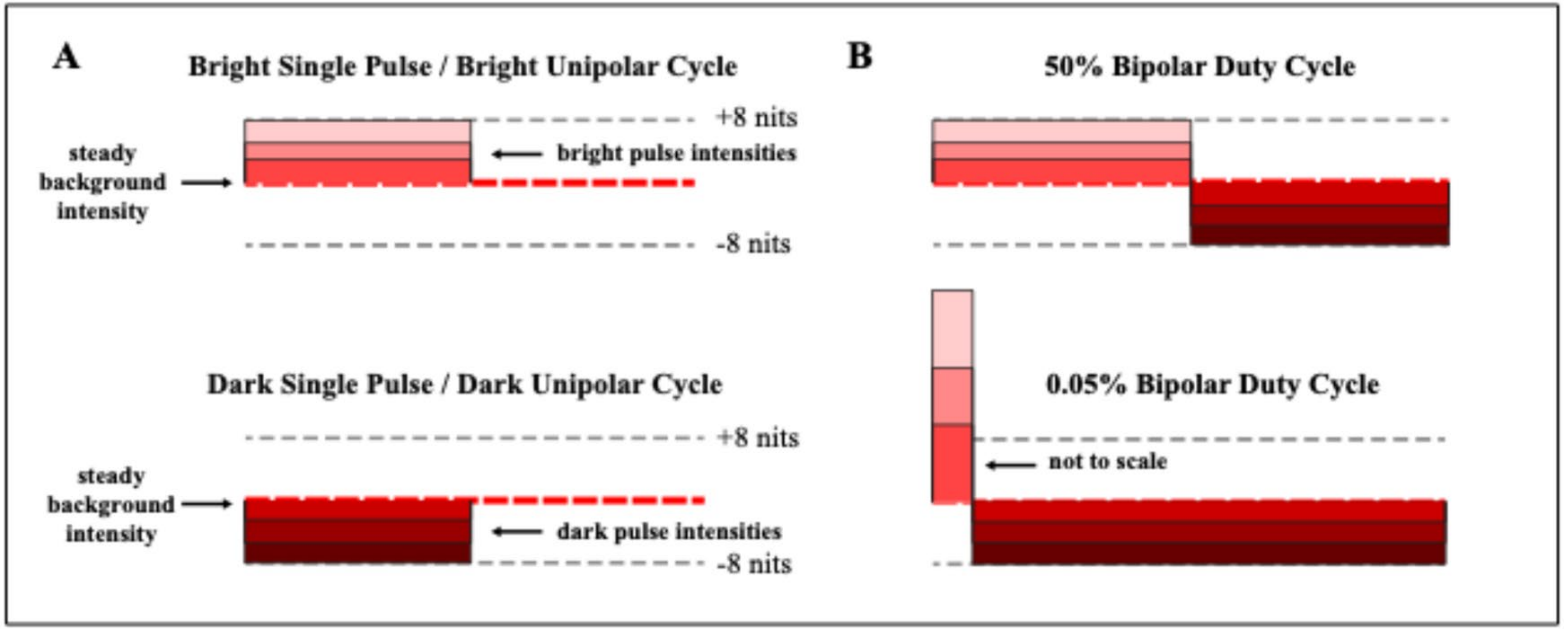
(**A**) Single-pulse tasks provided a dark or bright pulse with a specified duration and intensity on each trial. Unipolar flicker tasks provided a dark or bright pulse for half of each flicker cycle, with emission returning to background for the remainder of the cycle. (**B**) Bipolar tasks provided a dark and bright pulse within each flicker cycle, with various combinations of intensity and pulse duration.

Each trial presented a single letter with one of three methods: single pulse, unipolar flicker, or bipolar flicker. Single-pulse tasks displayed the letter with one dark or bright pulse that had a specific duration and intensity. Unipolar flicker tasks provided dark or bright pulses with a specified intensity, a duration that was half the cycle period, with a return to background emission for the remainder of the period. Bipolar flicker tasks provided a dark and a bright pulse within each period, with various combinations of intensity and duration. These three conditions are illustrated in Fig. 2.

Pulses were scaled relative to the light energy that was provided by the background. Light energy is defined as the product of intensity and duration (nits x µs). For example, a bright pulse that is twice the intensity of background and lasts for 10,000 µs has a departure energy of 80,000 nit-µs (8 nits above background multiplied by 10,000 µs). Dark energy is a reduction of emission from background. Note that dark pulses have a cap on the maximum amount of energy reduction, in that departures cannot go below 0 emission. This limits the range of energies that can be evaluated with dark pulses, so all of the present work adopted this as the maximum energy departure for both polarities.

For single-pulse tasks, energy was scaled with respect to the duration of the pulse itself, 100% being the maximum that could be delivered by a dark pulse. For flicker tasks, energy was scaled with respect to the duration of the period, 100% being the maximum that could be delivered if the dark pulse filled the full period.

It is convenient to describe the size of the increments and decrements in absolute terms, e.g., a 25% dark pulse would reduce background emission by 25%, and a bright pulse would increase emission by the same amount. However, for labeling of figures we may specify the dark and bright pulse energy with minus and plus values, respectively.

### Single-pulse tasks

#### Task 1: recognition of letters as a function of single pulse energy and polarity

All pulses of a given test session were either dark or bright, with pulse amplitude being varied from trial to trial. Pulse duration was 10,000 µs. Pulse energy was varied from 10% to 100% in 10% increments. With 10 levels of pulse energy, 2 levels of pulse polarity, each combination being repeated 20 times, there were 400 total display trials for a given test session. Letter recognition data were provided by one group of five respondents.

#### Task 2: bright pulse duty-cycle across five orders of magnitude

Bright pulse durations were varied across five orders of magnitude, specifically at 1, 10, 100, 1000, and 10,000 µs durations. Pulse energy was varied across a range that produced a linear change in recognition probability in Task 1, specifically with pulse energy from 26.09% to 44.35% of background energy. This energy range was sampled at 20 equal increments, and for a given sampled level, pulse intensity was adjusted to maintain the same pulse energy for each of the five pulse durations. The combinations comprised of 5 durations x 20 energy levels were each repeated 4 times for a total of 400 trials for a given test session. Letter recognition data were provided by one group of ten respondents.

#### Task 3: confirming correspondence of recognition for 10 µs pulses

Each trial displayed a letter with a 10 µs bright pulse energy range from 0% to 100%. Except for the use of an ultrabrief pulse duration, all other conditions were the same as Task 1. Letter recognition data were provided by one group of five respondents.

### Unipolar flicker tasks

#### Task 4: dark and bright unipolar flicker at 50, 100, and 200 Hz

The unipolar flicker of Task 4 provided either a dark pulse or a bright pulse for half of each cycle, with emission returning to the background level for the remainder of the cycle period. Letters were displayed at frequencies of 50, 100, and 200 Hz. The corresponding cycle period for those frequencies are 20,000, 10,000, and 5,000 µs. Pulse energy was specified as a percentage of background energy, which for these and subsequent flicker tasks was across the cycle period. With the pulses being half of a given period, the maximum energy that could be provided by dark flicker was 50%. Although bright emission is not capped in this way, bright flicker was tested with this being the maximum energy level. Flicker energy varied from 5% to 50% in 5% increments. Each trial displayed flicker for 128 cycles. Each treatment level was repeated 40 times for a total of 400 total trials. The three frequencies were tested with each polarity having its own group of five respondents.

#### Task 5: letter recognition as a function of number and duration of flicker cycles

Task 4 found that letter recognition rose to an asymptote just below 100%, with the transition occurring at about 25% energy for each of the three frequencies. Dark and bright flicker manifested similar levels of activation. Task 5 provided this 25% energy level for each letter display and varied the number of cycles from 1 to 128 (in eight octave steps) to assess the summation of pulse influence. A zero treatment condition was added, wherein letters were not displayed on the 0 trials. This provided a better anchor for the 50 Hz condition, in that pilot work found that just one cycle could produce a substantial amount of recognition. In addition, the 0 treatment level provided for consistent modeling of frequency effects where recognition was plotted against display time rather than number of cycles. The nine levels of cycle number were tested in combination with the three frequency levels, each combination being replicated 15 times for a total of 408 trials. The dark and bright combinations were judged by two separate groups of five respondents.

### Bipolar flicker tasks

#### Task 6: Energy balance varying frequency and duty cycle

The bipolar flicker of the present report displayed dark and bright pulse pairs that filled each cycle. Energy of dark and bright pulses was restricted to 0–25% of background energy across the full flicker cycle, which was found to be the effective range for producing zero to near 100% recognition in Task 4. Energy levels for dark and bright pair members were a combination of fixed and variable amounts -- one member of the pair being fixed at 25% energy and the other with energy that varied from 0% to 25% in 5% increments. So half of the displays provided dark flicker at −25% energy, with the bright flicker being at one of the 0–25% levels. The other half provided bright flicker at + 25% energy, with the dark flicker being at one of the 0–25% levels.

Duty cycle was a major treatment condition of Task 6, this being an adjustment of the duration of dark and bright pulses. For 50% duty cycle the pair members had the same duration, each occupying half of the cycle period. For 0.05% duty cycle, duration of bright pulses were reduced by four orders of magnitude and dark pulses expanded to fill the remainder of the cycle period. For the 50, 100, and 200 Hz displays, bright pulse durations were 2.5, 5, and 10 µs, and corresponding dark-pulse durations were 2497.5, 9995, and 19,990 µs, respectively. Intensities for dark and bright pair members were adjusted to maintain the energy being specified for each pair combination.

Recognition of the bipolar combinations was evaluated for 200, 100, and 50 Hz flicker, these treatments being displayed at either 50% or 0.05% duty cycle. As a minor control treatment, flicker was presented either with the dark pulse leading or following the bright pulse. The duty cycle, and pulse order conditions were tested with four separate groups of respondents, five per group, with the expectation that pulse-order data would be combined if initial modeling did not find meaningful differentials in performance. For each of the sub-tasks, a given trial displayed the bipolar pair for 128 cycles, and each treatment combination was repeated 20 times, providing 400 total trials.

#### Task 7: Higher-range energy balance for frequency and duty cycle

Task 7 replicated the conditions of Task 6, but with a higher range of pulse energy. Half of the trials provided the fixed dark-pulse member with 50% energy with the other half emitting 50% energy for the fixed bright-pulse member. Variable energy ranged from 25% to 50% in 5% increments, with each of the variable levels being paired to the fixed energy level of the opposite polarity. The pulse energy and frequency combinations were tested with 11 replications for a total of 398 total trials. The two levels of duty cycle were tested with separate groups of five respondents.

#### Task 8: Full-range energy balance for frequency with 0.05% duty cycle

Task 8 examined recognition probabilities for 50, 100, and 200 Hz frequencies, with displays that provided fixed pulses with up to 100% energy. Half of the trials provided the fixed dark-pulse member with 100% energy and the other half emitted 100% energy for the fixed bright-pulse member. Variable energy ranged from 10% to 100% in 10% increments. For pulses delivering pulse energy up to 100%, duty cycle become moot, in that one must derive the energy above 50% by increasing the duration of the dark pulse to fill the full cycle. As the dark-pulse durations reaches 99.95% of the period duration, it provides the dark component of a flicker at 0.05% duty cycle. A given trial displayed the bipolar pair for 128 cycles, with each treatment combination being repeated 20 times for a total of 400 trials. Each frequency was judged by a separate group of five respondents.

### Experimental approval and execution

Experimental protocols were approved by the USC Institutional Review Board, whose guidelines include requirements for informed consent to be tested, and the ability to discontinue participation at any time without recourse. The guidelines include safeguards for comfort of the respondent, as well as privacy, confidentiality, and anonymity. We adhered to the guidelines and regulations in the conduct of this research.

Eighty-five respondents were recruited from the Psychology Subject Pool. Each provided judgments for only one of the tasks described above. There were 25 males and 60 females, with ages ranging from 18 to 22. Informed consent was obtained from each respondent. They were told of task requirements, and that they could discontinue participation at any time without penalty. Visual acuity was tested, but the small amount of variation did not appear to be a factor in task performance.

The test room was illuminated by a single 7 W DC bulb (6000 K) that provided 10 lx of steady ambient light. The bulb was positioned on the wall above where the respondent was seated, at 2.25 m from the floor. These lighting conditions had previously been found to produce an average pupil diameter of 6.66 mm.

The LED display was tangent to the line of sight of respondents at a distance of 1.5 m, and respondents viewed the display with both eyes open. Background luminance of the display was maintained throughout the session. On a given trial a letter was randomly chosen from the alphabet, and was displayed with a treatment combination that was randomly chosen. A brief tone accompanied the letter display in case the letter was imperceptible. Respondents were expected to name the letter or say that no letter had been perceived. For some tasks the respondents also reported whether the letters flickered, and whether they appeared bright or dark. The experimental conditions were not designed to provide reliable assessment of these judgments, and they were included as pilot measures, i.e., to inform subsequent work that might yield useful data. The experimenter launched successive trials and entered the responses on the computer. Neither the respondent nor the experimenter was given any feedback about whether letters were correctly identified. A session was normally completed in about 45 min.

### Statistical methods

All statistical modeling was performed in Python using the *statsmodels*,* pwlf*, and *pygam* libraries. Unless noted below, 95% confidence intervals (CIs) were obtained from each package’s built-in methods.

Across tasks, a given model estimated the probability of letter recognition as a function of an independent treatment variable, e.g., pulse energy or number of display cycles. Responses were binary, with each trial coded as 1 for recognition and 0 for non-recognition. The estimated probability of recognition at each treatment level was derived from the aggregate of binary responses across all trials and participants for that task.

Piecewise linear regression was applied to single-pulse Task 1 and Task 3. A three-segment (two breakpoints) model was fit to recognition probability versus pulse energy. Breakpoint locations were estimated with nonlinear least-squares optimization that minimized RSS (Residual Sum of Squares) via the L-BFGS-B algorithm with multiple randomized initializations. This approach imposed boundary constraints and ensured that breakpoints were determined objectively, without manual adjustment or visual tuning. The confidence interval for each piecewise linear regression model was obtained with a nonparametric bootstrap with 1,000 resamples. Ordinary least-squares linear regression was used for Task 2, modeling recognition rate as a function of pulse energy.

Logistic regression was fit to unipolar-flicker Task 4 and Task 5. The number of cycles in Task 5 spanned six octaves, and the corresponding durations ranged from 0 to 2560 milliseconds. Therefore, a logarithmic transformation of the treatment variable was applied to compress the large dynamic range and better capture the underlying trend.

Logistic Generalized Additive Models (Logistic GAMs) were fit to bipolar-flicker Tasks 6 to 8, allowing flexible smooth models of recognition probability as a function of energy-balance predictors. All smooth terms used thin-plate splines with fine-tuned smoothing parameters.

The models for each of the tasks have been provided again in Supplemental Figures (These can be found through the Supplemental Information link at the end of the article), with the addition of tokens that mark the empirically observed group mean probability value at each treatment level. Each plot also reports the number of respondents who judged the task (contributed the data) and the corresponding fit statistics, i.e., R² and RMSE for linear and piecewise linear regressions; R² and AIC for logistic regressions and generalized additive models (GAMs).

## Results

### Single-pulse tasks

#### Task 1: comparing bright and dark departures from background with single pulses

The first task examined the degree to which the bright and dark channels are alike in response to energy level. With a background intensity set at 8 nits, the task displayed a randomly chosen letter with either a 10,000 µs bright pulse or a 10,000 µs dark pulse, with levels of departure that ranged from 10% to 100% of background energy across the pulse duration.

**Fig. 3:**
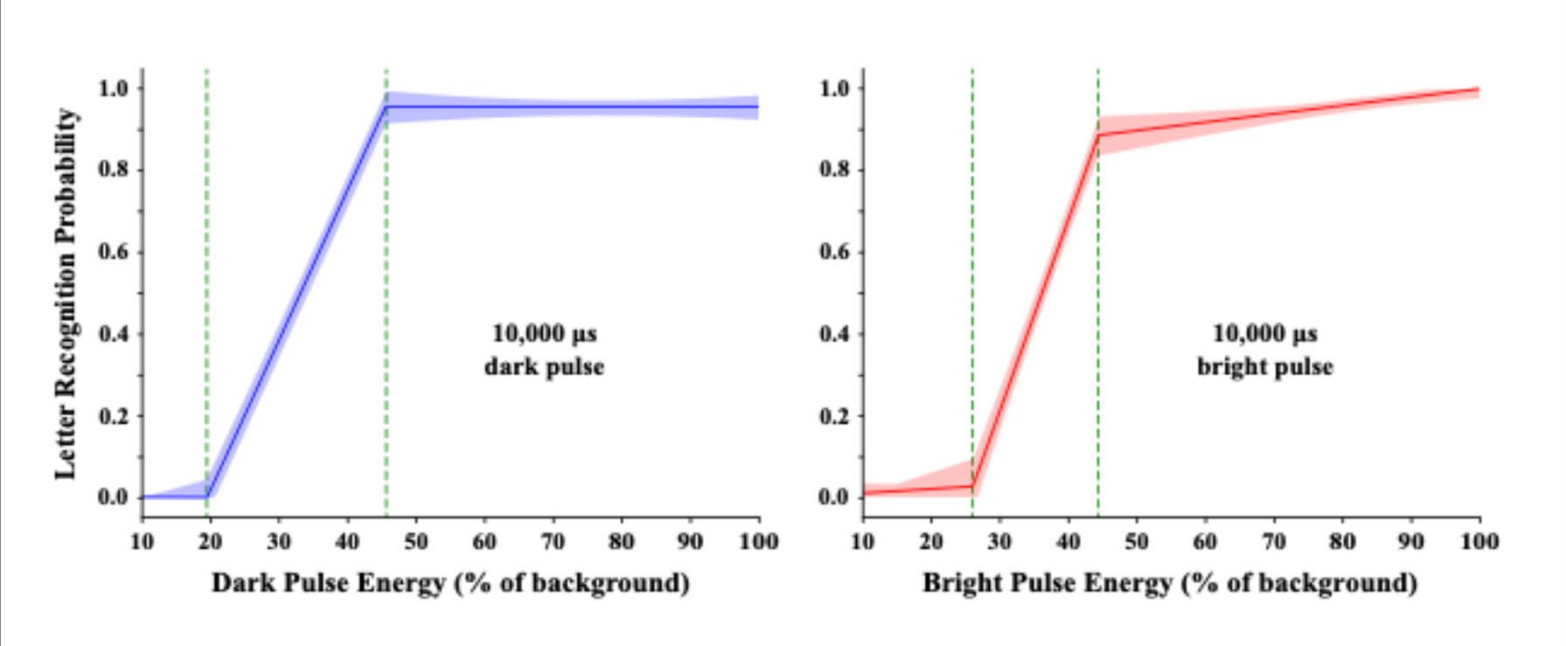
Each trial of the first task displayed a randomly selected letter with a single dark or bright pulse, with pulse energy ranging from 10–100% of background energy. Piece-wise linear regression provided the best fitting models, with a central component that provided a steep linear rise. The dark and bright models were similar in terms of the range and slope of the linear rise in probability of recognition.

Initial examination of the raw means suggested three discrete stages of letter recognition -- virtually no recognition at the lowest energy levels, a steep rise at mid-levels, and a relatively flat asymptote. Formal linear modeling confirmed this observation for bright pulses and dark pulses alike, these models being provided in Fig. 3. Vertical dash lines have been added to delineate the central region that shows a steep linear rise in letter recognition. The energy range for the bright and dark models differ a bit, and the probability of letter recognition reaches a slightly higher level for the dark model. But overall the dark and bright departures from background were very similar in their ability to elicit letter recognition.

We think it is noteworthy that probability of recognition rises as a linear function of stimulus energy. Recognition is mainly determined by relative brightness of the letters, which is specified by Talbot-Plateau calculations that require linearity of component values. It is also interesting that the linear range was sharply capped at 90–95% recognition, this being elicited by less than 50% of the energy that could have been delivered by a single pulse. This might well reflect the operating range of a population of retinal neurons that register contrast and thus provide for visibility of the letters. We expect that the operating range depends on the energy level of the background.

#### Task 2: consistency of pulse energy as a function of pulse duration

It would be useful to establish whether a reduction in pulse duration changes the ability of pulse energy to elicit letter recognition. One can test this across several orders of magnitude with bright pulses by increasing pulse intensity while proportionately reducing pulse duration. The same isn’t available for dark pulses, at least not for the full linear range that one can see in Fig. 3, because one cannot reduce pulse intensity to less than 0 nits (zero emission).

**Fig. 4:**
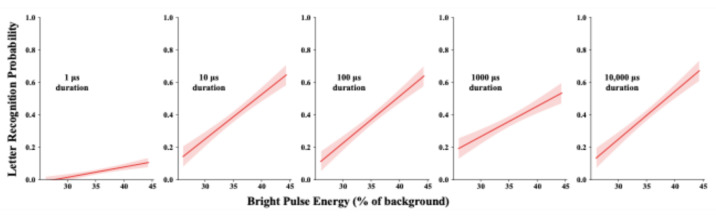
Each letter was displayed with a single bright pulse that had durations ranging from 1 µs to 10,000 µs. Pulse energy was varied using the same range that produced a strong linear rise in recognition in Task 1, this being accomplished by increasing intensity proportionally as a function of pulse duration. The resulting models of recognition as a function of pulse energy were comparable, except for a greatly reduced effect for 1 µs pulses.

Task 2 provided trials with pulse durations at 1, 10, 100, 1000, and 10,000 µs, i.e., from 0.05% to 50% duty cycle. Display trials sampled the range of pulse energy that had produced the strong linear rise in letter recognition (delineated by dashed lines in Fig. 3). Levels within that range were multiplied as needed to provide equivalent pulse energies for each of the tested durations. Linear models of letter recognition by a second group of respondents is shown in Fig. 4.

The results of Task 2 indicate fairly comparable ability of pulse energy to elicit recognition for durations that range from 10 µs to 10,000 µs. None of the models rose as much as was found in Task 1. The 10,000 µs model failed to reach the upper levels of recognition, even though the display conditions on those trials was the same as for Task 1. Perhaps the mixed complement of pulse duration affected responsiveness of retinal mechanisms. That issue is examined in Task 3 that follows shortly.

A major difference can be seen for displays that used 1 µs bright pulses. The model still manifested a linear rise in probability of letter recognition, but with the energy being much less effective. Recognition was only 10% at energy levels that produced 90% recognition when the pulses were displayed for 10,000 µs. Apparently one has reached a physiological limit on response to the pulse energy. This is not to say that 1 µs pulses are incapable of eliciting letter recognition; earlier articles from this laboratory have documented that they can do so^22,23^. One presumes that to elicit much higher recognition, a 1 µs display must be brighter than what was provided in this task.

#### Task 3: replicating task 1 using 10 µs pulses

**Fig. 5:**
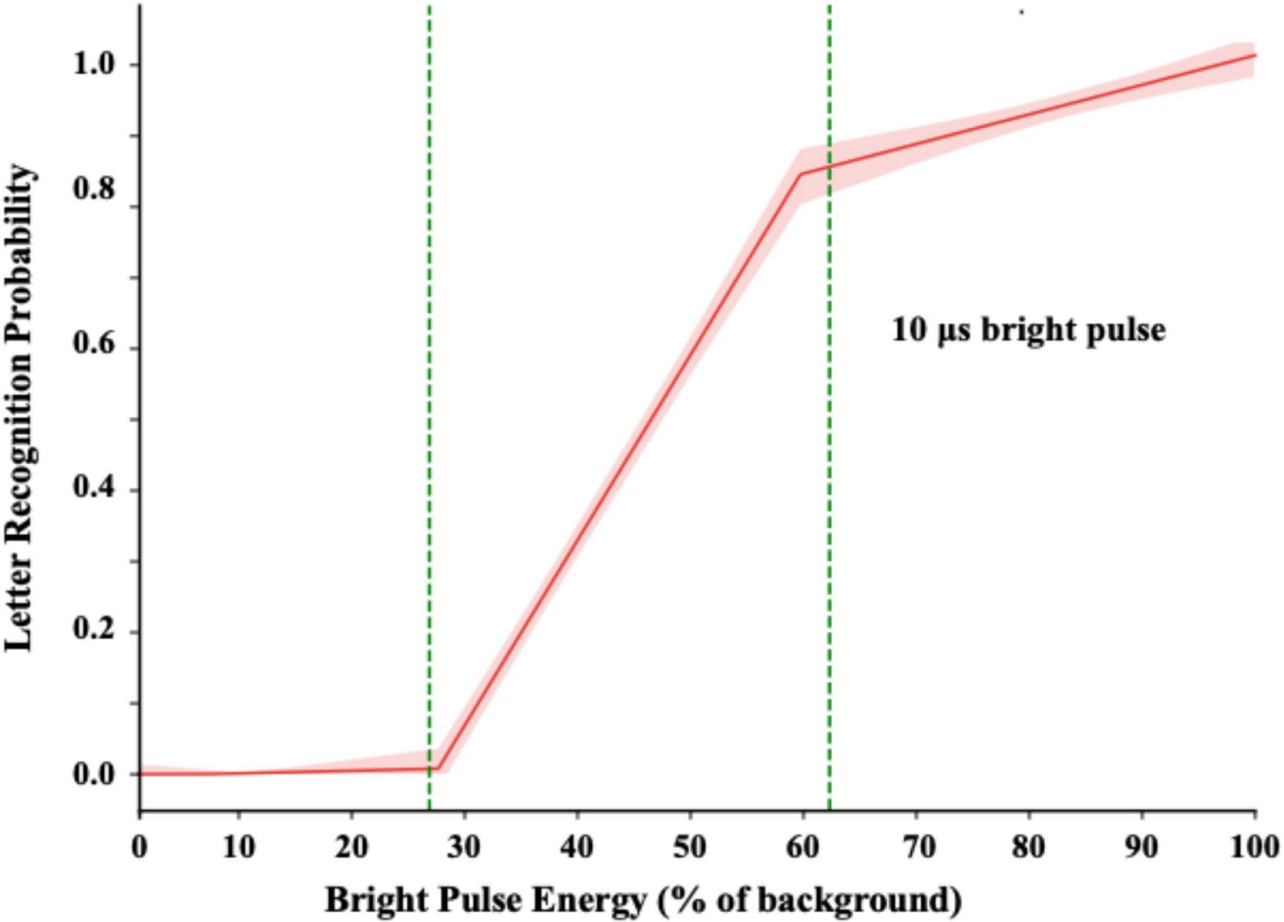
Task 3 evaluated whether display of letters with 10 µs bright pulses would elicit recognition that was comparable to what was produced with 10,000 µs bright pulses. The linear range is somewhat wider than was found for 10,000 µs bright pulses, but the basic structure of the two are very similar.

The duration manipulations of Task 2 found energy effects that were linear, but with the probability of recognition rising less than was observed in Task 1. Perhaps this was due to the greater number of durations that were displayed during the session. There might be adaptation mechanisms that register pulse energy, providing greater precision of response where a given energy level is consistently repeated. Requiring a response to various levels during the same session might not provide the same precision. To see if this might be the case, Task 3 displayed only 10 µs bright pulses, with all conditions other than duration being identical to the first task. Figure 5 provides the model of these judgments.

The activation slope for Task 3 was less steep than for Task 1, but the probabilities of recognition that could be elicited were almost identical. The upper break-point for recognition was in the 90% range for both. We can infer that the pulse energy needed for recognition of letters ranges from roughly 20% to 50% of background energy, that the rise is linear, and it is comparable for pulse durations of 10 µs and 10,000 µs.

### Unipolar flicker tasks

#### Task 4: bright and dark unipolar flicker at 50, 100, and 200 Hz

Task 4 displayed pulse sequences that were either dark or bright departures from background, with flicker frequency being 50, 100, or 200 Hz. For a given frequency the duration of each pulse was half the period, the remainder of a given period being a return to background emission. These sequences are characterized as “unipolar flicker,” with further descriptions being “dark flicker” and “bright flicker.” Energy of a given flicker was again a percentage of background energy, with 100% being the background energy across the full cycle period, not across the duration of the pulse (as in Tasks 1–3).

**Fig. 6:**
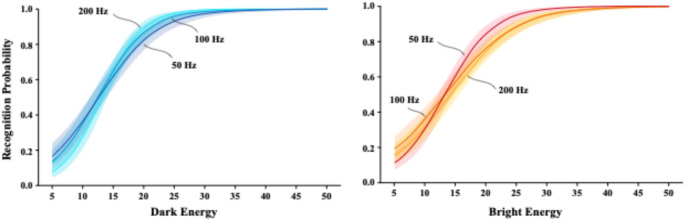
The left panel provides logistic regression models for dark unipolar flicker for each of the three frequencies as a function of stimulus energy. The right panel provides the corresponding models for bright flicker. Dark and bright alike reached asymptotic levels of letter recognition with stimulus energy of roughly 25–30% of background energy.

Figure 6 shows logistic regression models for the six task combinations, with the dark flicker models for the three frequencies being shown in the left panel and the bright flicker models being shown on the right. All six models were very similar in how stimulus energy affected probability of recognition. For dark and bright flicker alike, there was a steep rise in recognition from 10% energy to 25%, transitioning to a flat recognition asymptote just below 100% across all other energy levels. Performance was very close to the same for dark and bright flicker at each of the frequencies, supporting the inference that dark and bright retinal channels are balanced in registering pulse energy.

Probability of recognition for each of the three frequencies became asymptotic at about 25% energy. Collapsing data across dark and bright models above that energy level, mean recognition was 93, 96, and 97% for 50, 100 and 200 Hz, respectively. Could this be a classic “ceiling effect,” wherein the activation being provided by the stimulus exceeds the maximal response that can be rendered, i.e., reliable recognition of letters? Or is it a “cap” on an operating range, like what was found for single-pulse displays? The data are quite consistent in providing confidence bands that lie just below 100% across all the sampled energy levels for each of the six models, i.e., dark and bright x three frequencies. If the activation were simply exceeding the maximal response that could be registered, one would expect to see the confidence band disappear into the ceiling at higher levels of flicker energy.

Further, statistical evidence can be derived by collapsing data across the 25–50% range of flicker energy. When this was done for each of the six models, the smallest and largest mean difference from 100% recognition were 5.2 and 6.9 standard-error units. Some would report the significance for all of those outcomes as *p* < 0.000. For individual models as well as treatment categories, the evidence clearly indicates that this recognition ceiling is just below 100% for all flicker energy levels at and above 25% of background energy. We infer that this a cap on the activation range for a specific population of retinal neurons that are designed to register contrast against the level of background that was used.

#### Task 5: varying the number of unipolar flicker cycles

In Task 4, letters were displayed with dark and bright unipolar flicker at 50, 100, and 200 Hz, and with pulse energy at 25%. That level of energy was delivered for 128 cycles on every trial. Task 5 varied the number of cycles to assess how quickly the activation develops for each of the frequencies, and again establish whether dark and bright channels had a balanced response. The number of cycles ranged from 1 to 128, but the protocol added a 0 condition (no letter being displayed), so that modeling of recognition could be rendered as a function of time in addition to number of cycles.

**Fig. 7:**
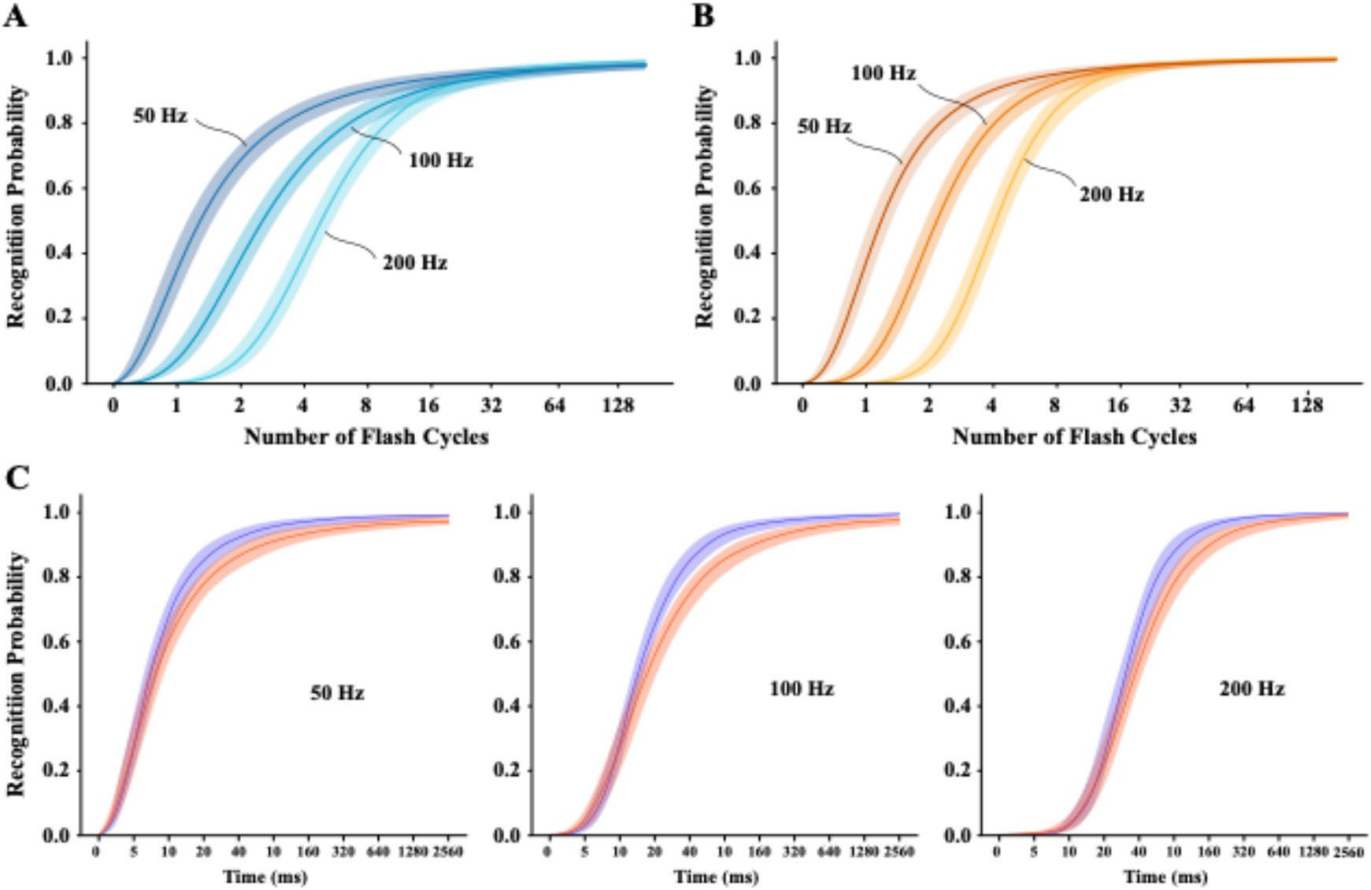
The upper panels show models of the rise in recognition for each of the three frequencies as a function of the number of display cycles. Models for dark flicker are provided in panel A, and the corresponding models for bright flicker as shown in panel B. The lower panels show the dark and bright models for each of the three frequencies separately, with conversion of the number of cycles to milliseconds. When scaled as time, the rise in recognition for the three frequencies is fairly similar.

Logistic models for Task 5 can be seen in Fig. 7. Panels A and B show that recognition as a function of cycle number was about the same for dark and bright flicker, and models for all three frequencies rose to asymptotes that were below 100%. Models for dark and bright flicker were very similar. The rise in recognition began progressively later as frequency was increased, and the rate of rise was steeper the lower the frequency. With only one cycle there was recognition of almost half the letters with 50 Hz displays. The total amount of energy being delivered per second was the same for all three frequencies, so we infer that the sustained activation that was provided by longer duration pulses was responsible for the differential rate of rise.

The models converge at just under 100% recognition by 32 cycles, and maintain that level across all remaining cycle levels. Mean recognition levels at 32 cycles were 95, 93, and 94% for the 50, 100, and 200 Hz models, respectively. Figure 7C provides models for dark and bright flicker at each of the frequencies, with cycle number being converted to display time. These panels affirm that there was very little difference in the activation provided by dark or bright flicker, but also that each frequency reached the asymptote with roughly the same display time, i.e., in about 160 ms. So a unipolar flicker stimulus that is displayed for 160 ms or more, with differentials in frequency from 50 to 200 Hz, does not produce differentials in recognition.

Asymptote level remained just below 100% recognition for each of the three frequencies, similar to what was seen in Task 4 (Fig. 6). Combining across asymptotic energy levels, mean separation from 100% recognition was significant for all six polarity by frequency combinations, with the smallest and largest being 11.6 and 27.8 standard-error units. Again we suspect that this reflects an operational cap on channel signaling rather than a classic ceiling effect.

### Bipolar flicker tasks

#### Testing in the 0–25% energy range

Task 6 used bipolar combinations of pulse energy that were within the range of energy that produced a monotonic rise in probability of letter recognition in unipolar flicker Tasks 4 and 5. Each display paired a fixed 25% dark pulse energy against six levels of bright pulse energy from 0 to 25% to provide differentials in the dark/bright energy balance. The reverse condition paired a fixed 25% bright pulse energy with the corresponding six levels of dark pulse energy. The combination that provided 25% energy levels for both dark and bright pulses had balanced energy levels, which were expected to provide brightness that matched background. Each combination was displayed at 50 Hz, 100 Hz, and 200 Hz, and across two levels of duty cycle (50% and 0.05%).

**Fig. 8:**
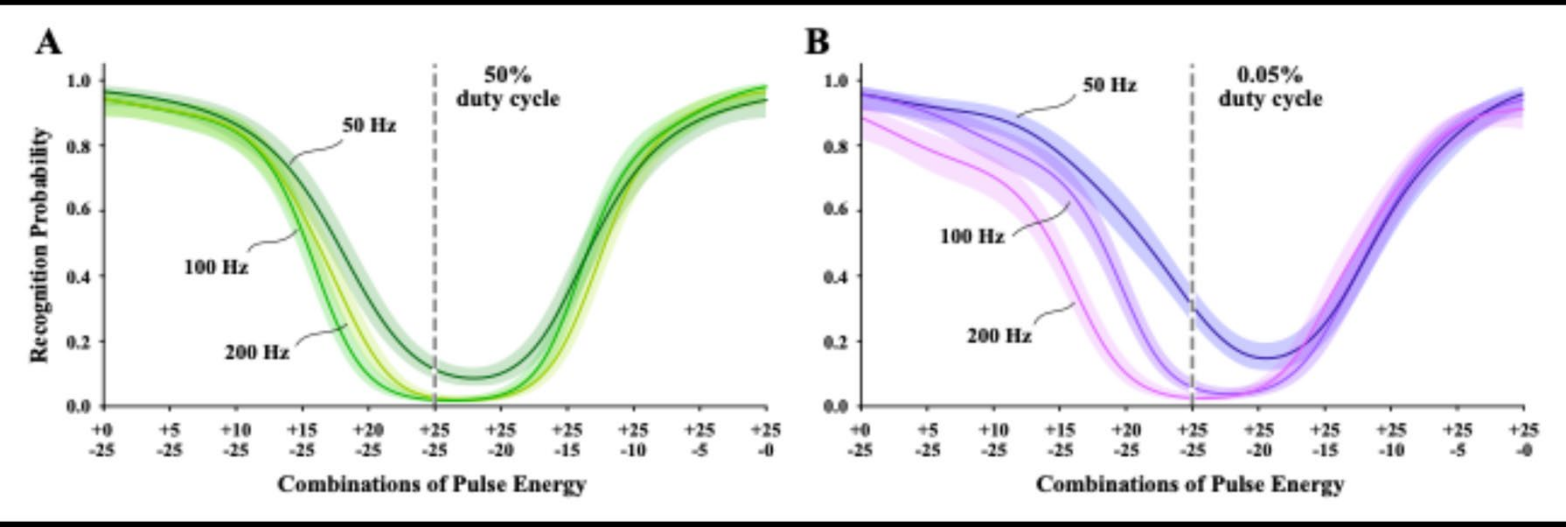
The two panels provide logistic models for 50, 100, and 200 Hz bipolar flicker, wherein each cycle displays a dark pulse combined with a bright pulse. Labels of pulse energy levels for each combination specify the energy of the bright pulse above and the dark pulse below. In each panel, the combination that provided equal and opposite amount of energy are marked with a dashed vertical line. Displays providing judgments to the left of this line were weighted with a progressively larger proportion of dark energy, and displays on the right had progressively larger bright-energy weights. (**A**) Models for 50% duty cycle manifested reduction of letter recognition to near zero as the dark and bright energy weights reached equal energy balance, which was the expected result. (**B**) The 0.05% duty cycle models manifested larger recognition differentials. But the reduction in duty cycle did not produce the anomalous contrast effects that had previously been found with low duty-cycle displays.

Subgroups were tested with displays in which the dark-pulse came before, or after, the bright-pulse member. There was no indication that order of pulse polarity produce differential effects on letter recognition, so these subgroups were combined for modeling the treatment combinations. Figure 8A shows the probability of recognition as a function of the dark/bright energy weights for 50% duty cycle. Consistent with prior work^20,21^, letter recognition was near zero for each frequency where the pulse combination was balanced, i.e., where the combination provided + 25% and − 25% energy levels. Model profiles were very similar for 50, 100, and 200 Hz displays, as predicted by the Talbot-Plateau law. However, minimum recognition for the 50 Hz model was somewhat higher than for the other two frequencies, and the minimum for all three models was shifted somewhat to the right. At present we have no explanation for either of these differentials.

To the left of the balance point the letters were displayed with 25% dark pulses being paired with progressively lower bright energy levels, and recognition rose to almost 100% as the bright energy declined to zero.

energy declined to zero. The reverse was found on the opposite side where 25% bright pulses were paired with progressively lower dark-pulse energy. Again, this was consistent with prior work^21^.

Figure 8B shows recognition plotted against the polarity weight levels for letters displayed with 0.05% duty cycle. The three frequencies manifested larger differentials in letter recognition than was seen with 50% duty cycle, and the model minimum for 50 Hz displays was markedly shifted to the bright side of energy balance. However, what is most notable was a lack of much anomalous contrast effect. As previously reported^20,21^, letter recognition was expected to be substantially boosted for displays done with 0.05% duty cycle. Much of that work was done using 250 Hz bipolar displays, but we have found the anomalous-contrast effect at frequencies ranging from 25 to 250 Hz^24^.

#### Testing in the 25–50% energy range

The combinations of Task 6 used the 0–25% energy range that has produced a steep rise in letter recognition in Tasks 4 and 5. Perhaps the 25–50% range, wherein dark pulses can reach maximal departure from background -- zero emission -- would elevate recognition when displayed at 0.05% duty cycle.

**Fig. 9:**
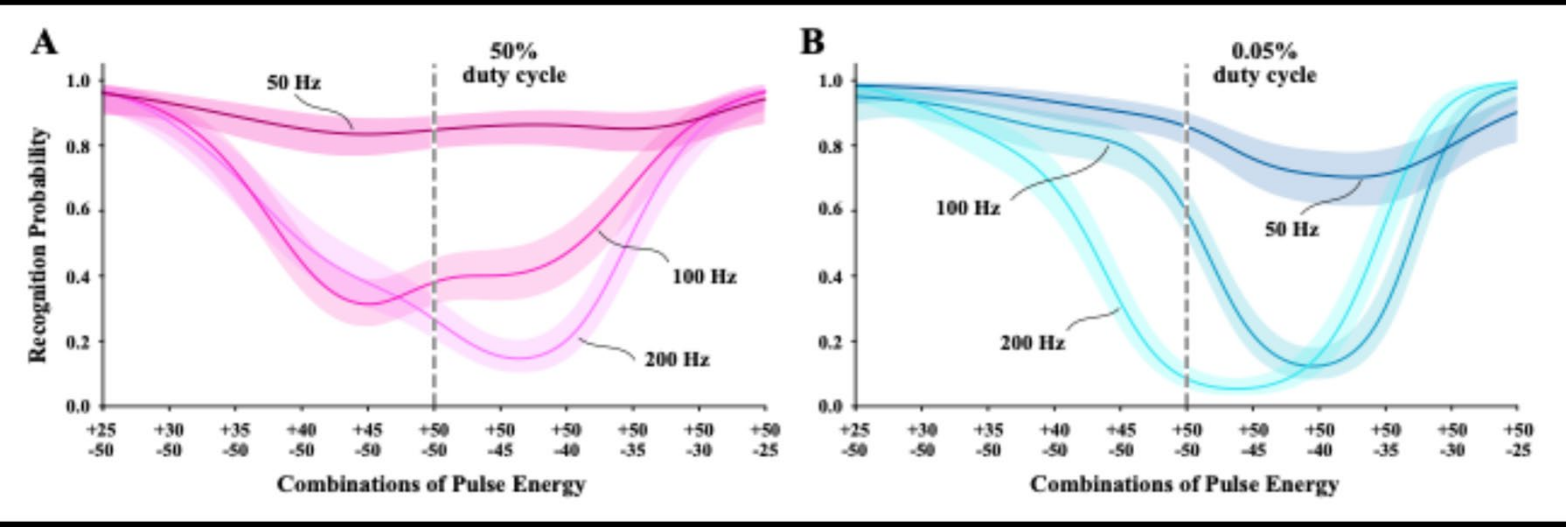
Task 7 asked for displays using energy levels in the 25–50% range to see if these conditions would provide anomalous contrast. (A) The upper-energy levels of Task 7 produced some elevation of recognition for 100 and 200 Hz treatment combinations, and raised recognition into the 90–100% range for the 50 Hz treatment combinations. (B) The 0.05% duty cycle produced substantial asymmetry in the models of recognition for each of the three frequencies. Letter recognition was not substantially elevated at 100 and 200 Hz. These results do not suggest strong and reliable anomalous contrast effects.

Task 7 provided fixed dark-pulse energy at 50% for half the trials and fixed bright-pulse energy at 50% for other trials of a given session. The variable energy pulses ranged from 25 to 50%, pairing fixed and variable pulses having opposite polarity. Figure 9A provides models for the three frequencies that were displayed at a 50% duty cycle, and Fig. 9B shows the 0.05% models.

Using combinations in the energy range from 25% to 50% clearly affected probability of letter recognition. The 50 Hz models were elevated into a much higher probability of recognition for both levels of duty cycle -- 50% as well as 0.05%. It is possible that this reflects the action of anomalous contrast, even though it has not previously been found at 50% duty cycle. The 50 Hz frequency is just above the flicker-fusion threshold, below which the Talbot-Plateau principles that call for averaging of dark and bright energies does not apply. It is possible the flicker-fusion threshold itself varies depending on the energy range being used to produce flicker.

Models of 100 Hz and 200 Hz displays that were done with 50% duty cycle had higher probabilities of recognition than were found for 0.05% duty cycle. This is the reverse of what would be expected for an anomalous-contrast effect. The minimum probability of recognition was shifted to the right for most of the models. This suggests greater influence of dark pulses, which might be due to differentials in the relative strength of OFF and ON retinal channels. This matter will be discussed subsequently.

**Fig. 10:**
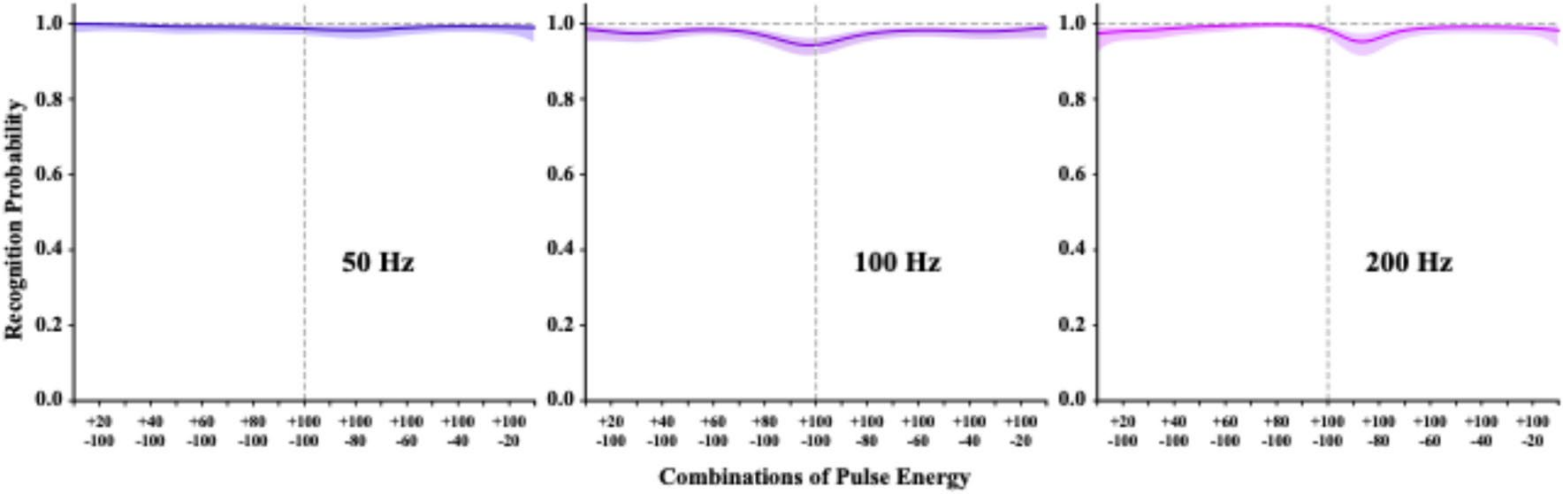
The models of letter recognition for maximum pulse-energy combinations are provided for 50, 100, and 200 Hz flicker. Anomalous contrast elevated letter recognition across all combinations of pulse energy, including for combinations that provided 100% energy to both the dark- and bright- flicker components.

#### Testing in the 10–100% energy range

Task 7 provided combinations wherein the maximum dark-pulse energy was 50%, which means that dark pulses provided zero emission for displays at 50% duty cycle. But the energy is being specified across the full period, i.e., zero emission for only half the cycle equals 50% pulse energy. A 0.05% duty cycle increases dark-pulse duration to 99.95% of the cycle period (bright pulses providing the remaining 0.05%), and the extended duration requires a reduction in pulse intensity. So using the 25–50% energy range did not produce energy levels that were comparable to earlier work that produced the reported anomalous contrast effects. To do that, the dark-pulse intensity would need to be maximal for almost the entire duration of the cycle, which would deliver a pulse energy that was 99.95% of background. For simplicity of discourse, this will be described as a 100% energy level.

Task 8 used a 10–100% energy range that approximated treatment combinations that had been shown to produce anomalous contrast^20,21^. Half of the trials displayed fixed dark-pulse energy at 100% and the other half displayed fixed 100% bright-pulses energy. The variable pulses provided energy ranging from 10% to 100%, in 10% steps, with variable pulses at each level being paired with fixed pulses having opposite polarities. The models based on judgments of these combinations are provide in Fig. 10, using separate panels to display the models for 50, 100, and 200 Hz frequencies.

For each of the three tested frequencies, letter recognition was near 100% across all combinations of pulse energy, including the central balance point where the dark/bright energy levels matched, i.e., with 100% dark-energy pulses being paired with 100% bright-energy pulses. The merge with background that rendered the letters invisible in Task 6 did not occur. This agrees with prior work that found elevated probability of letter recognition for letters displayed with 0.05% duty cycle, which we have described as an anomalous contrast effect^20,21^.

## Discussion

A major goal was to establish what probability of letter recognition will be produced as a function of energy level of dark and bright pulses. Energy of a given pulse was scaled as a percentage of background energy. The first single-pulse task found that letter recognition was below threshold for recognition when pulse energy was below 20–25% of background energy. Beyond that transition, increased energy produced a linear rise in probability of recognition until an upper transition just below 50% energy for dark and bright pulses alike (see Fig. 3). Increased energy beyond the upper transition produced no increase in recognition for dark pulses, and a much slower rise in recognition for bright. The range and rate of rise in recognition probability was comparable for dark and bright displays. The second and third tasks demonstrated that reducing bright pulse duration by four orders of magnitude (with adjustment of intensity to maintain the same level of pulse energy), produced a near identical rise in probability of letter recognition as was found in the first task (see Figs. 4 and 5). This is consistent with the Talbot-Plateau law.

For this task, we can describe pulse energy below 50% of background energy as being in a “linear range,” and the transition level can be thought of as a “cap” on this range. However, the energy levels above the high transition point are linear as well, though at a dramatically lower slope. Burkhardt and associates described responses of salamander bipolar cells as being linear with weak stimulus intensities with a transition to nonlinear responding with stronger stimulation^25^. A sharp change in the amount of energy required to change a cell’s response might well be interpreted as transition from a linear to nonlinear activation.

However, we are reluctant to accept characterization of the linear range as a weak stimulus. The test room was dim, and at 8 nits the background appeared very bright. In other work that used twice that intensity, respondents complained that the array was too bright for comfort. Clearly the levels of dark and bright pulse energy was sufficient for producing reliable scaling of letter recognition, so we are not providing stimulation that is near the perceptual threshold. Our results clearly demonstrate comparable (symmetrical) processing of dark and bright pulses. Very likely, studies reporting asymmetrical sensitivity to dark and bright stimuli were using energy levels that were above our linear range.

Unipolar flicker is produced when each cycle period displays only a dark pulse or bright pulse, with a return to the background light level for the remainder of the period. The present work examined dark and bright unipolar flickers having frequencies (number of pulses per second) of 50, 100, and 200 Hz. Here the pulse energy was specified relative to the duration of the period, unlike single-pulse displays where the energy was specified relative to the duration of the pulse.

As can be seen in Fig. 6, increases in pulse energy produced something close to a linear rise in probability of letter recognition, with a transition to asymptote at about 25% of background energy. Respondents manifested greater variability in the energy at which this transition occurred, therefore we modeled the data using logistic regressions rather than linear regression. One can see in Fig. 6 that dark and bright unipolar flicker produced a similar rise in probability of recognition as a function of pulse energy for each of the three frequences. Probability of recognition was asymptotic just below 100% for each frequency, as detailed above.

For Task 5 the number of cycles was varied, and the three frequencies differed substantially the number of cycles required to produce corresponding levels of letter recognition. However, the frequencies were alike in reaching a common asymptote (again just below 100%) with display time of about 160 milliseconds. This suggests that probability of recognition is being controlled by (absolute) energy. A specific amount of net energy is needed to reach the signaling limit, and the rate at which one gets to that amount is a function of the duration of the pulse cycle. Each 50 Hz pulse lasts 20 milliseconds, so it can reach a given level of aggregate absolute energy with fewer cycles. Likewise, 100 Hz and 200 Hz require progressively more cycles to provide the same energy. This new perspective on how letter recognition depends on an aggregate energy total can also explain the findings for Task 4 (Fig. 6).

Each cycle of bipolar flicker displayed both a dark and bright pulse. Each bipolar flicker task displayed various combinations of dark and bright energy with three levels of frequency (50, 100, and 200 Hz) and two levels of duty cycle (50% and 0.05%). Three ranges of dark and bright pulse energy were used in Tasks 6, 7, and 8, specifically 0–25%, 25–50%, and 50–100%, respectively.

For Task 6, each display presented a combination that had a 25% energy pulse of one polarity paired with a pulse of the opposite polarity which could vary in energy level from zero to 25%. This made it possible to see how reducing the weight of one polarity relative to the other would affect letter recognition. A major goal was to replicate prior findings that the 50% duty cycle would provide models consistent with the Talbot-Plateau law and the 0.05% duty cycle would provide models with elevated letter recognition, i.e., producing anomalous contrast^20,21^.

Figure 8 A shows that letters displayed with 50% duty cycle were generally consistent with Talbot-Plateau predictions. Recognition for the combinations at or near energy balance for dark and bright pulses (−25/+25) were in the chance range for 100 Hz and 200 Hz displays. The 50 Hz frequency was a little above chance, suggesting a minimal activation of anomalous contrast mechanisms. Minimum recognition was shifted somewhat to the right of the predicted balance point for each of the frequencies, which will be discussed below.

The models for 0.05% duty cycle (Fig. 8B) were also shifted to the right and minimum recognition for 50 Hz displays was elevated a bit more. Otherwise, the models were generally consistent with the Talbot-Plateau predictions. These were not the results that were expected. Based on earlier work, the expectation was a high probability of recognition for all three the frequencies for all pulse combinations, including where the energies of dark and bright pulses were equally balanced.

The prior work that demonstrated anomalous contrast effects had specified the intensity levels of dark and bright pulses, and we suspected that using energy units (intensity x duration) to specify treatments was the reason for the unexpected outcome. The maximum Task 6 energy levels were only 25% of background energy across the cycle period, so even with dark duration being 99.95% of the period, the dark intensity was well below 100% departure from background intensity. Tasks 7 and 8 were designed to evaluate the pulse combinations with energies in the 25–50% and 50–100% ranges.

The models of Task 7 (Fig. 9) demonstrate that providing combinations with energy levels above the linear range did produce anomalous contrast effects, for recognition was elevated for each frequency and across duty cycles. The 50 Hz displays manifested the strongest anomalous contrast. Elevation of recognition probability was less with 100 Hz and 200 Hz displays, and the models were less alike than was found with Task 6 (Fig. 8).

The use of combinations with 25% to 50% energy levels produced the greatest asymmetry of letter recognition models. This suggests that the reports of differential in perception of dark and bright stimuli that were noted in the Introduction might be due to using luminance levels that are above the optimal range for processing of image information. Alternatively the asymmetry effects might show up at the boundary for selecting retinal subpopulations that are designed to register and signal different contrast ranges.

For Task 9 (Fig. 10), which was done with maximum energy levels, recognition was at or near 100% at each frequency (50, 100, and 200 Hz) across all bipolarity combinations. This shows that one can produce effective anomalous contrast by providing large pulse-energy differentials, i.e., those that are above 50% of background energy. This finding would support the hypothesis that the range of contrast provided by the stimulus determines which retinal subpopulation will be activated.

## CODA

The single-pulse tasks provided an energy range, wherein the increments of pulse energy produced linear increments in the probability of letter recognition. This suggests that the predictions of the Talbot-Plateau law that normally are applied to flickering stimuli also pertain to single-pulse displays. The Talbot-Plateau law specifies what combination of (frequency x duration x intensity) will equal the brightness of a steady display. For the single pulse task, frequency is not relevant, but the (duration x intensity) portion of the calculation is a specification of pulse energy. The model with 10 us pulses was almost identical to the model for 10,000 us pulses, which confirms the reciprocity of the Talbot-Plateau calculation. The basic message is that the Talbot-Plateau principle applies to single-pulse activation of bright-channel mechanisms (with the frequency component having a value of 1). Presumably it would apply just as well to the dark-channel mechanisms with suitable consideration of emission limits, i.e., not being able to deliver pulses less than zero emission.

It is interesting that recognition as a function of pulse energy was “capped” for single-pulse displays at a high probability of recognition, and for unipolar flicker at just below 100%. This suggests application of an efficient encoding principle that links responsiveness of the signal source to the judgment that makes use of that signal. That control is likely being set by synaptic influence from horizontal cells. It is generally accepted that horizontal cells register the average luminance that surrounds bipolar cells, and provide adjustments for the level of ambient light^26–31^. As applied to the present findings, the background energy would be registered by the horizontal cells, providing surround influence on the receptive fields of bipolar cells. This influence might provide a limit on the activation of bipolar cells.

There is substantial variation from cell to cell in contrast sensitivity, and the distribution of these response variables matches the statistics of natural images^13,25,32^. Jiang and associates studied activity of OFF and ON channels in monkeys as they performed a contrast detection task and noted responses that matched natural scene statistics^33^. Hsiang and associates used two-photon calcium imaging in mouse to monitor synaptic terminals of ON bipolar cells and found that temporal and spatial information for artificial and natural images produced comparable activations^34^. The general suggestion is that subpopulations of bipolar cells can register contrast differentials within a specific range, with horizontal cells determining which subpopulation is allowed to speak^35^.

## Supplementary Information

Below is the link to the electronic supplementary material.

Supplementary Material 1

Supplementary Material 2

## Data Availability

Subject logs for each of the tasks are available at the Figshare repository, doi 10.6084/m9.figshare.29826983.
