# Supplementary material for: Balance and imbalance in dark and bright (OFF and ON) visual channels
Source: Sci Rep. 2026 Jan 14;16:1764. doi: 10.1038/s41598-025-28463-y (PMC12804859; doi:10.1038/s41598-025-28463-y)

Balance and Imbalance in Dark and Bright (OFF and ON) Visual Channels

Ernest Greene and Jack Morrison, Psychophysics Research Laboratory
Department of Psychology, University of Southern California
Los Angeles, California, United States

Supplemental Figures

Recognition choices were binary, so the probability of letter recognition for a given treatment level is based on the mean taken across numerous trials. The tokens that are plotted at treatment levels reflect the sum of the binary decisions across trials and across respondents.

Supplemental Figure 3

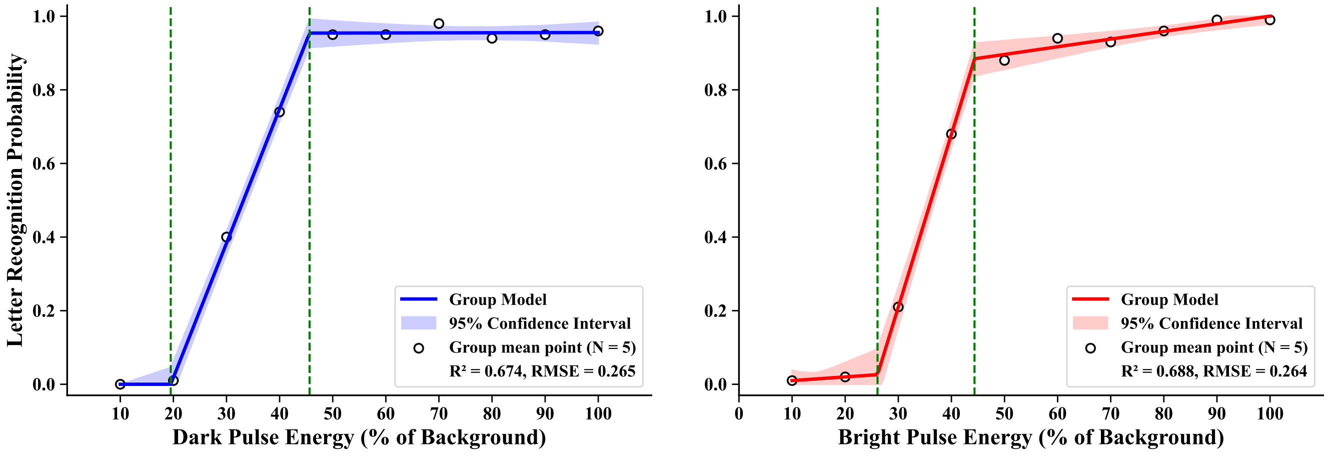

Supplemental Figure 4

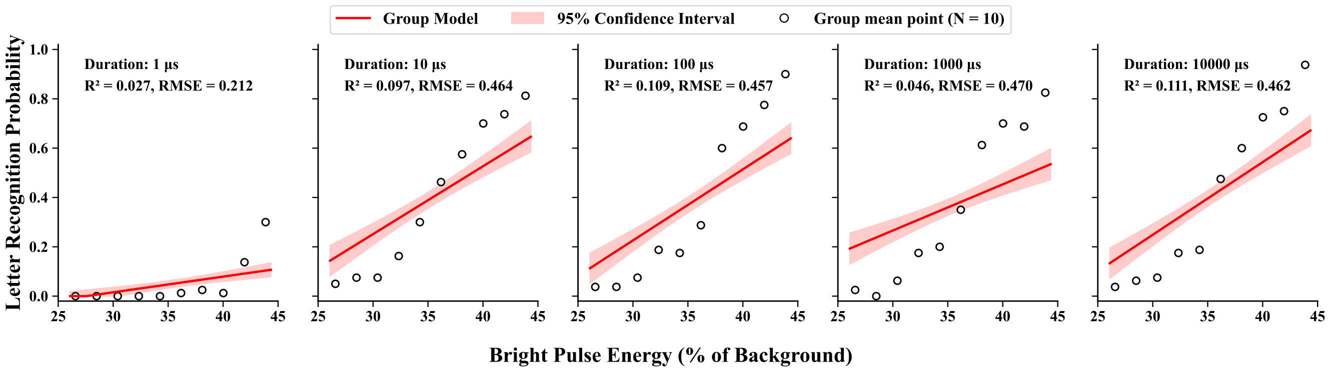

Supplemental Figure 5

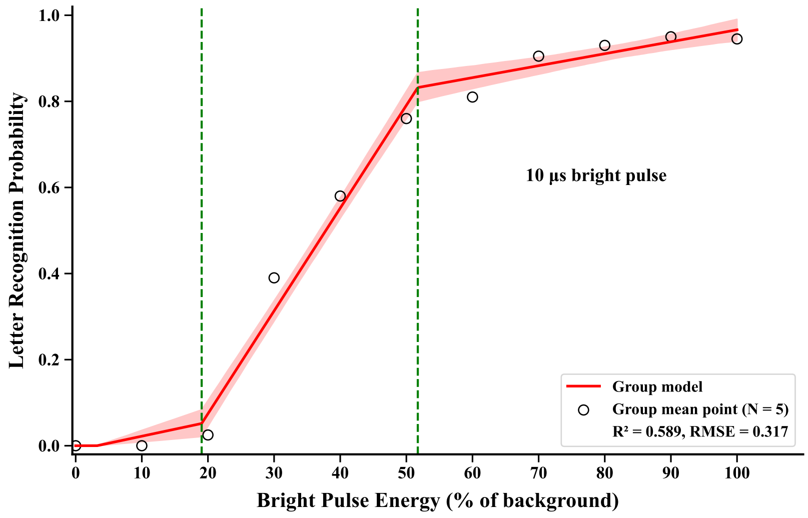

Supplemental Figure 6

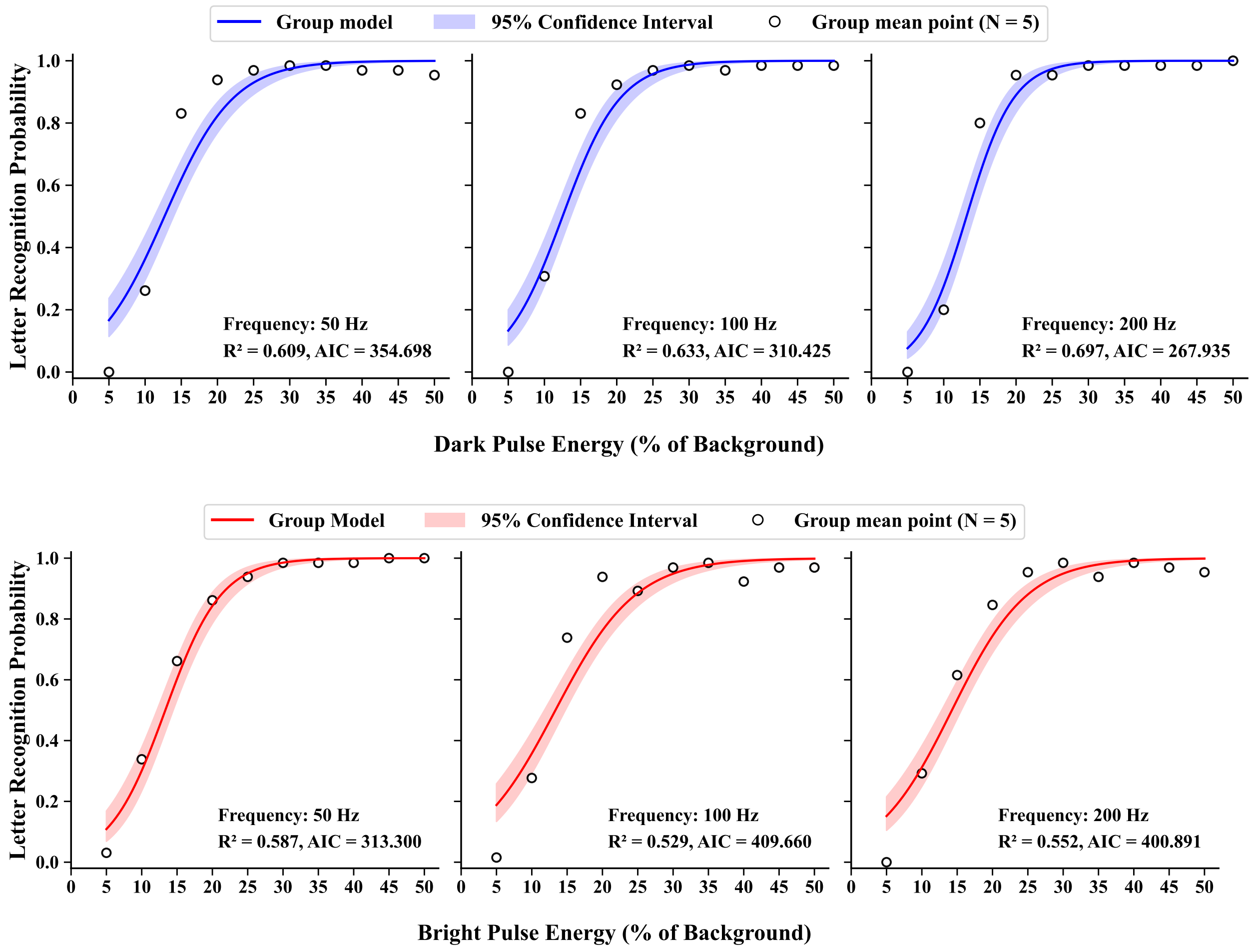

Supplemental Figure 7A and 7B

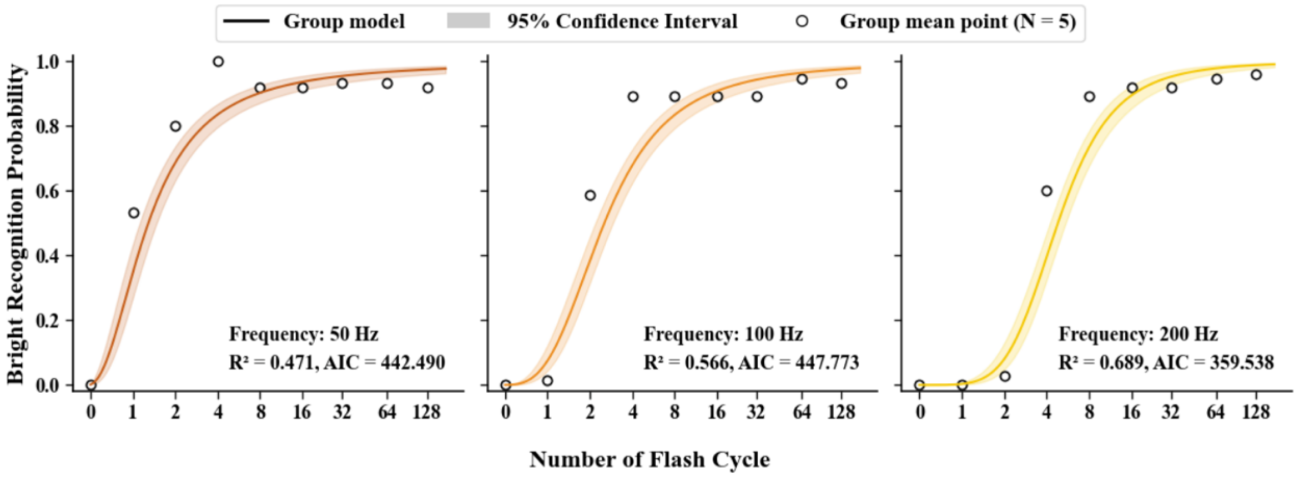

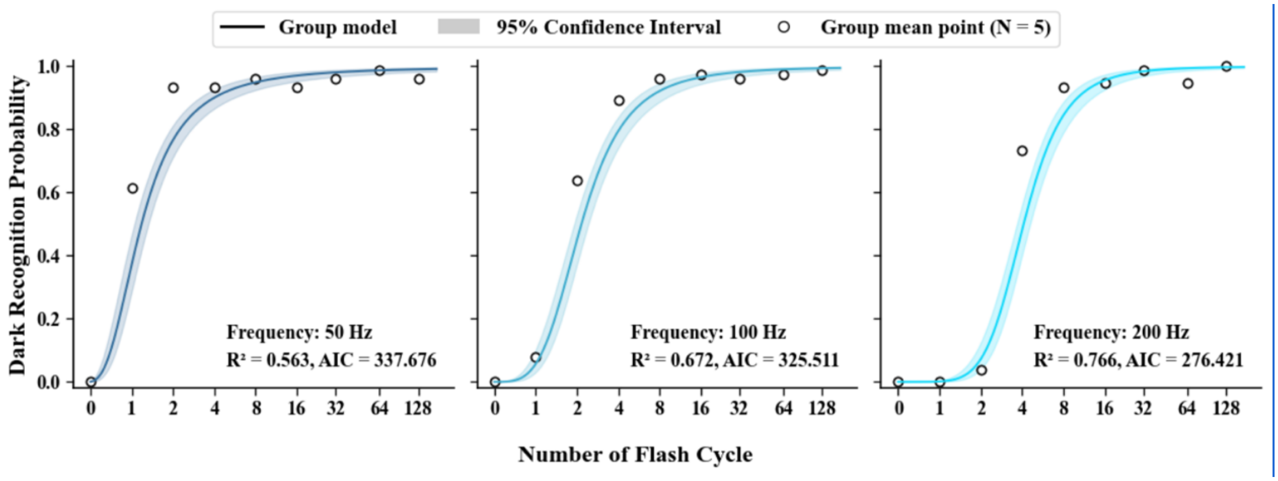

Supplemental Figure 7 C

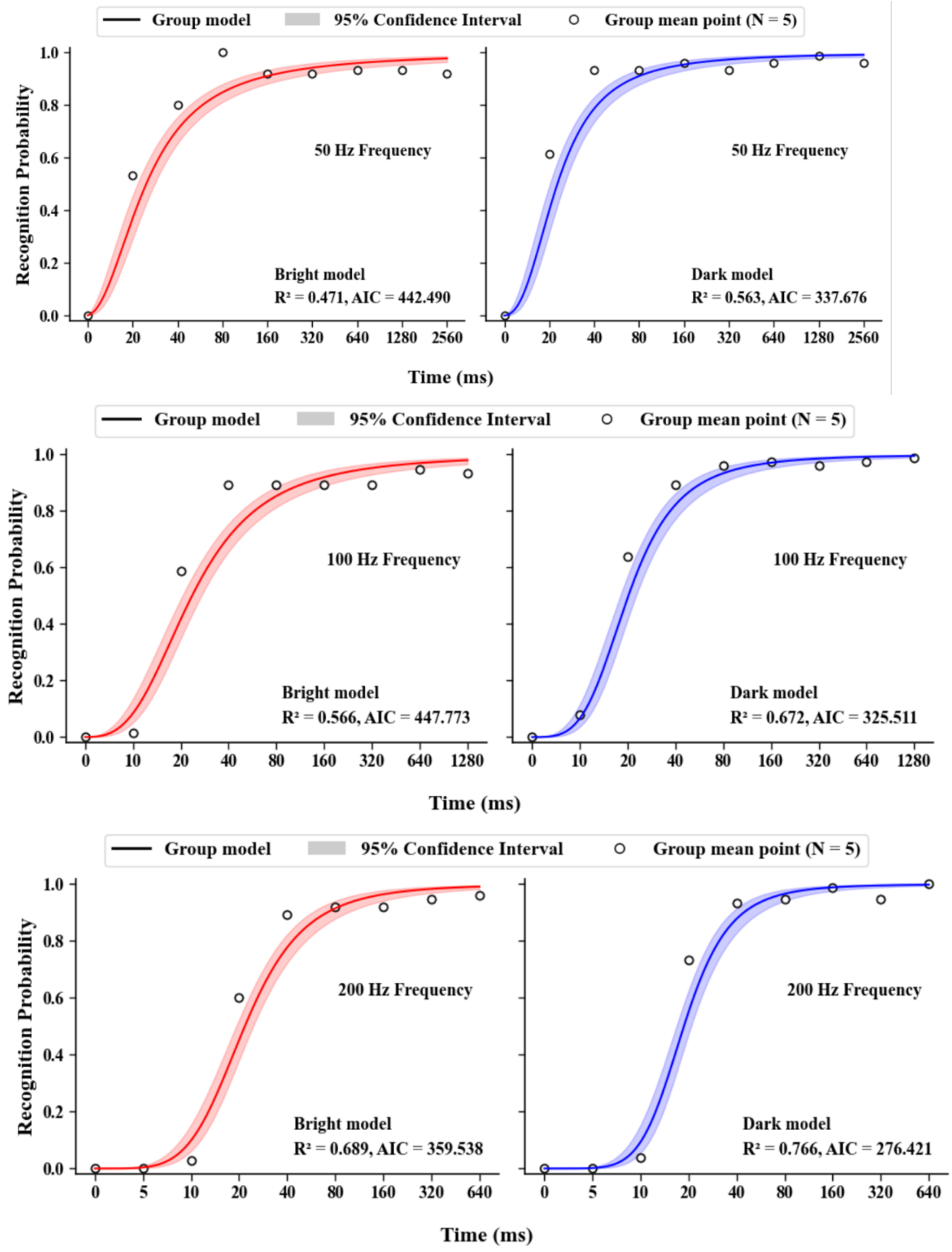

Supplemental Figure 8

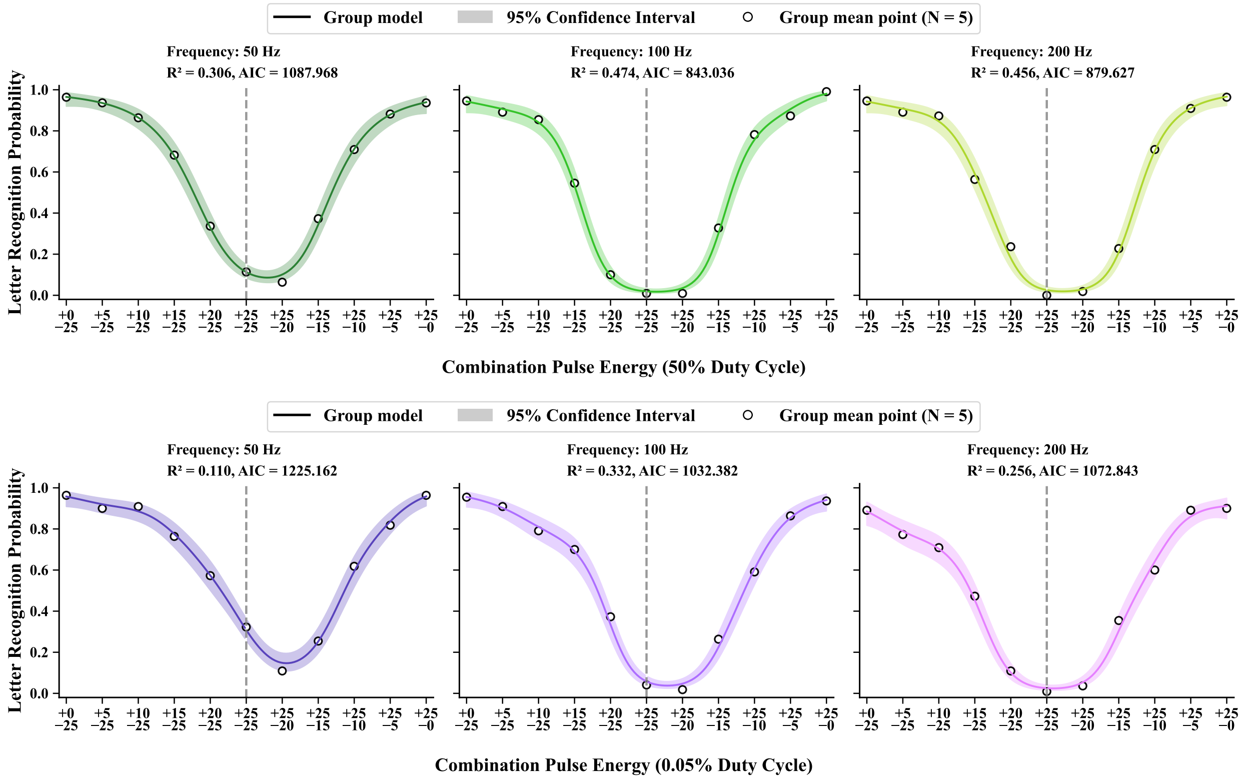

Supplemental Figure 9

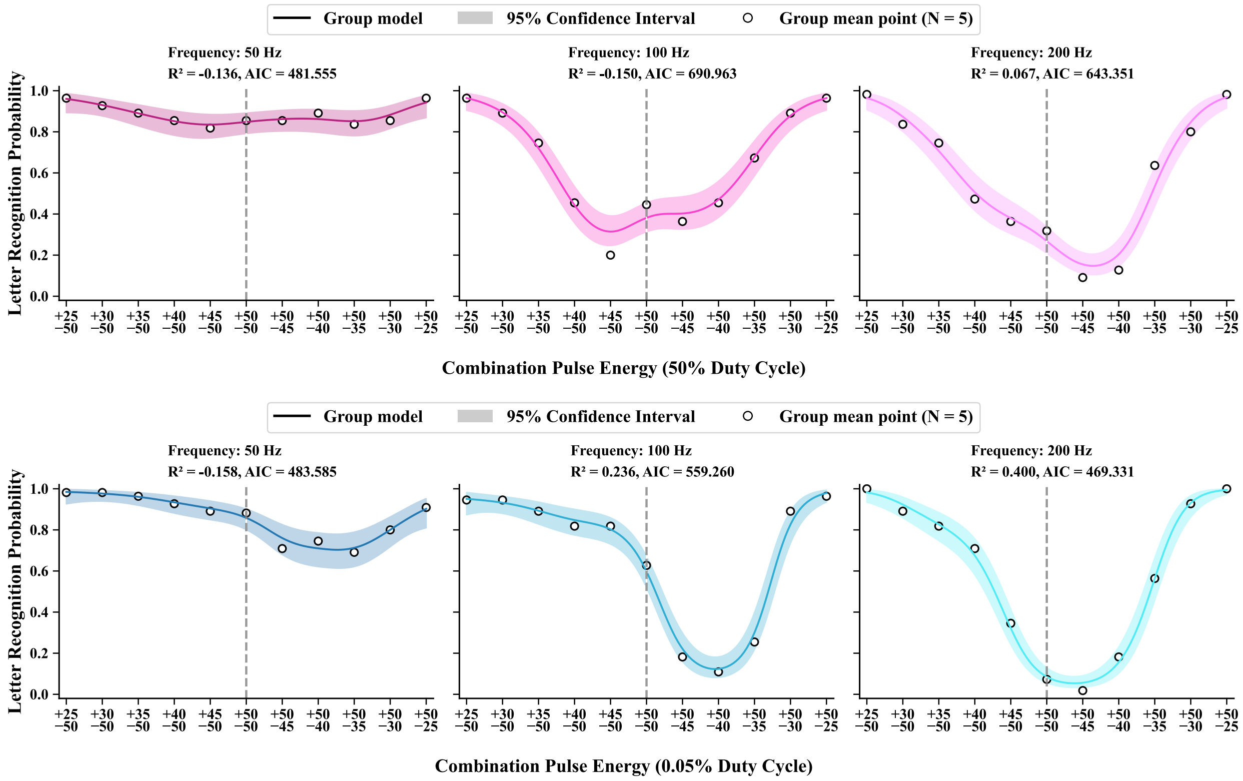

Supplemental Figure 10

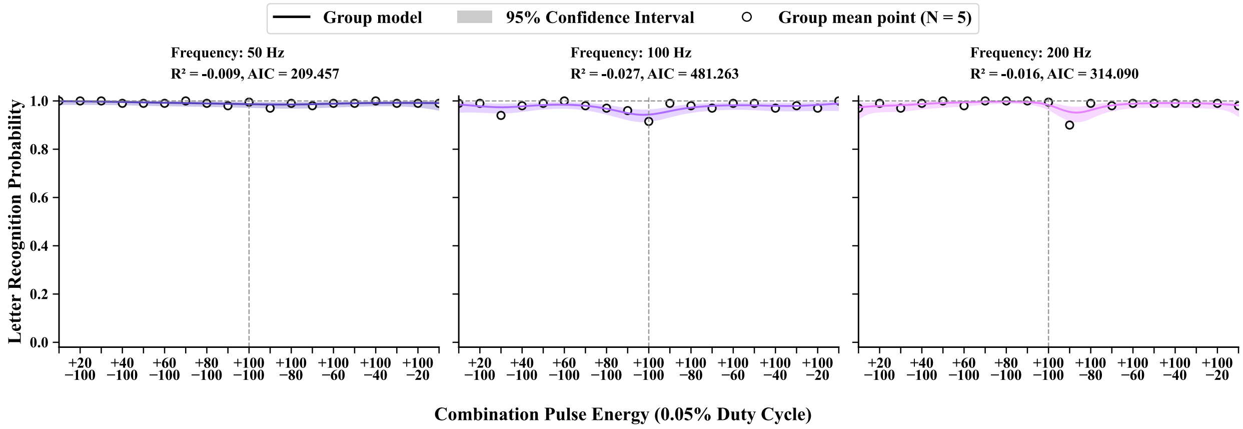

Supplement: Supplementary file 1 — Supplementary Material 1 [file 41598_2025_28463_MOESM1_ESM.pdf]
